# Supplementary material for: COZOID: contact zone identifier for visual analysis of protein-protein interactions
Source: BMC Bioinformatics. 2018 Apr 6;19:125. doi: 10.1186/s12859-018-2113-6 (PMC5889581; doi:10.1186/s12859-018-2113-6)
Supplement: Supplementary file 2 — Software build. Executable binary file of the software tool. (ZIP 118,784 kb) [file 12859_2018_2113_MOESM2_ESM.zip › cozoid/java/docs/beansbinding-1.2.1-doc.zip!/org/jdesktop/el/impl/ExpressionFactoryImpl.html]

ExpressionFactoryImpl (Beans Binding)


---


|  |  |  |
| --- | --- | --- |
| **Overview** \| **Package** \| **Class** \| **Use** \| **Tree** \| **Deprecated** \| **Index** \| **Help** | |  |
| PREV CLASS   **NEXT CLASS** | **FRAMES**    **NO FRAMES**     **All Classes** |  |
| SUMMARY: NESTED \| FIELD \| CONSTR \| METHOD | DETAIL: FIELD \| CONSTR \| METHOD |  |


---


## org.jdesktop.el.impl Class ExpressionFactoryImpl

```
java.lang.Object
  org.jdesktop.el.ExpressionFactory
      org.jdesktop.el.impl.ExpressionFactoryImpl
```

---

``` public class ExpressionFactoryImpl extends ExpressionFactory ```

**See Also:**: `javax.el.ExpressionFactory`

---

**Constructor Summary**

`ExpressionFactoryImpl()`


| **Method Summary** | |
| --- | --- |
| `java.lang.Object` | `coerceToType(java.lang.Object obj, java.lang.Class type)`             Coerces an object to a specific type according to the EL type conversion rules. |
| `MethodExpression` | `createMethodExpression(ELContext context, java.lang.String expression, java.lang.Class expectedReturnType, java.lang.Class[] expectedParamTypes)`             Parses an expression into a `MethodExpression` for later evaluation. |
| `ValueExpression` | `createValueExpression(ELContext context, java.lang.String expression, java.lang.Class expectedType)`             Parses an expression into a `ValueExpression` for later evaluation. |
| `ValueExpression` | `createValueExpression(java.lang.Object instance, java.lang.Class expectedType)`             Creates a ValueExpression that wraps an object instance. |

**Methods inherited from class java.lang.Object**

`clone, equals, finalize, getClass, hashCode, notify, notifyAll, toString, wait, wait, wait`

**Constructor Detail**

### ExpressionFactoryImpl

```
public ExpressionFactoryImpl()
```


**Method Detail**

### coerceToType

```
public java.lang.Object coerceToType(java.lang.Object obj,
                                     java.lang.Class type)
```

:   **Description copied from class: `ExpressionFactory`**
:   Coerces an object to a specific type according to the
    EL type conversion rules.

    An `ELException` is thrown if an error results from
    applying the conversion rules.

    :   **Specified by:**: `coerceToType` in class `ExpressionFactory`
    :   **Parameters:**: `obj` - The object to coerce.: `type` - The target type for the coercion.

---


### createMethodExpression

```
public MethodExpression createMethodExpression(ELContext context,
                                               java.lang.String expression,
                                               java.lang.Class expectedReturnType,
                                               java.lang.Class[] expectedParamTypes)
```

:   **Description copied from class: `ExpressionFactory`**
:   Parses an expression into a `MethodExpression` for later
    evaluation. Use this method for expressions that refer to methods.

    If the expression is a String literal, a `MethodExpression`  is created, which when invoked, returns the String literal,
    coerced to expectedReturnType. An ELException is thrown if
    expectedReturnType is void or if the coercion of the String literal
    to the expectedReturnType yields an error (see Section "1.16 Type
    Conversion").

    This method should perform syntactic validation of the expression.
    If in doing so it detects errors, it should raise an
    `ELException`.

    :   **Specified by:**: `createMethodExpression` in class `ExpressionFactory`
    :   **Parameters:**: `context` - The EL context used to parse the expression. The `FunctionMapper` and `VariableMapper` stored in the ELContext are used to resolve functions and variables found in the expression. They can be `null`, in which case functions or variables are not supported for this expression. The object returned must invoke the same functions and access the same variable mappings regardless of whether the mappings in the provided `FunctionMapper` and `VariableMapper` instances change between calling `ExpressionFactory.createMethodExpression()` and any method on `MethodExpression`. Note that within the EL, the ${} and #{} syntaxes are treated identically. This includes the use of VariableMapper and FunctionMapper at expression creation time. Each is invoked if not null, independent of whether the #{} or ${} syntax is used for the expression. : `expression` - The expression to parse: `expectedReturnType` - The expected return type for the method to be found. After evaluating the expression, the `MethodExpression` must check that the return type of the actual method matches this type. Passing in a value of `null` indicates the caller does not care what the return type is, and the check is disabled.: `expectedParamTypes` - The expected parameter types for the method to be found. Must be an array with no elements if there are no parameters expected. It is illegal to pass `null`. **Returns:**: The parsed expression

---


### createValueExpression

```
public ValueExpression createValueExpression(ELContext context,
                                             java.lang.String expression,
                                             java.lang.Class expectedType)
```

:   **Description copied from class: `ExpressionFactory`**
:   Parses an expression into a `ValueExpression` for later
    evaluation. Use this method for expressions that refer to values.

    This method should perform syntactic validation of the expression.
    If in doing so it detects errors, it should raise an
    `ELException`.

    :   **Specified by:**: `createValueExpression` in class `ExpressionFactory`
    :   **Parameters:**: `context` - The EL context used to parse the expression. The `FunctionMapper` and `VariableMapper` stored in the ELContext are used to resolve functions and variables found in the expression. They can be `null`, in which case functions or variables are not supported for this expression. The object returned must invoke the same functions and access the same variable mappings regardless of whether the mappings in the provided `FunctionMapper` and `VariableMapper` instances change between calling `ExpressionFactory.createValueExpression()` and any method on `ValueExpression`. Note that within the EL, the ${} and #{} syntaxes are treated identically. This includes the use of VariableMapper and FunctionMapper at expression creation time. Each is invoked if not null, independent of whether the #{} or ${} syntax is used for the expression. : `expression` - The expression to parse: `expectedType` - The type the result of the expression will be coerced to after evaluation. **Returns:**: The parsed expression

---


### createValueExpression

```
public ValueExpression createValueExpression(java.lang.Object instance,
                                             java.lang.Class expectedType)
```

:   **Description copied from class: `ExpressionFactory`**
:   Creates a ValueExpression that wraps an object instance. This
    method can be used to pass any object as a ValueExpression. The
    wrapper ValueExpression is read only, and returns the wrapped
    object via its `getValue()` method, optionally coerced.

    :   **Specified by:**: `createValueExpression` in class `ExpressionFactory`
    :   **Parameters:**: `instance` - The object instance to be wrapped.: `expectedType` - The type the result of the expression will be coerced to after evaluation. There will be no coercion if it is Object.class,


---


|  |  |  |
| --- | --- | --- |
| **Overview** \| **Package** \| **Class** \| **Use** \| **Tree** \| **Deprecated** \| **Index** \| **Help** | |  |
| PREV CLASS   **NEXT CLASS** | **FRAMES**    **NO FRAMES**     **All Classes** |  |
| SUMMARY: NESTED \| FIELD \| CONSTR \| METHOD | DETAIL: FIELD \| CONSTR \| METHOD |  |


---
